# Severity of coronary artery disease is associated with non-alcoholic fatty liver dis-ease: A single-blinded prospective mono-center study

**DOI:** 10.1371/journal.pone.0186720

**Published:** 2017-10-26

**Authors:** Mireen Friedrich-Rust, Fabian Schoelzel, Sebastian Maier, Florian Seeger, Julia Rey, Stephan Fichtlscherer, Eva Herrmann, Stefan Zeuzem, Joerg Bojunga

**Affiliations:** 1 Department of Internal Medicine 1, Hospital of the Goethe University, Frankfurt, Germany; 2 Department of Internal Medicine 3, Hospital of the Goethe University, Frankfurt, Germany; 3 Department of Medicine, Institute of Biostatistics and Mathematical Modelling, Goethe University Frankfurt, Frankfurt, Germany; 4 German Center for Cardiovascular Research, DZHK, partner site Frankfurt Rhine-Main, Berlin, Germany; Medizinische Fakultat der RWTH Aachen, GERMANY

## Abstract

**Background and aims:**

Liver steatosis has shown to be associated with coronary artery disease (CAD). The aim of our study was to evaluate the association between the presence and severity of CAD and Non-alcoholic fatty liver disease (NAFLD) assessed by transient elastography (TE) and controlled attenuation parameter (CAP).

**Methods:**

576 Patients undergoing coronary angiography were enrolled in this prospective study, receiving at least 10 TE and CAP measurements using the FibroScan® M-probe. Clinically relevant CAD (CAD 3) was defined as stenosis with ≥75% reduction of the luminal diameter. NAFLD was determined by CAP ≥234 dB/m. NAFLD with advanced fibrosiswas determined by TE-values ≥7.9kPa in the presence of NAFLD and absence of congestive or right-sided heart failure. Rates and 95% confidence intervals are shown.

**Results:**

505 patients were available for analysis of NAFLD. However, only 392 patients were available for analysis of NAFLD with advanced fibrosis, since 24 patients had to be excluded due to non valid TE-measurements and 89 patients due to congestive or right-sided heart failure or suspected concomitant liver disease, respectively. 70.5% (66.3%-74.4%) of patients had CAD 3, 71.5% (67.3%-75.4%) were diagnosed with NAFLD, and 11.2% (8.3%-14.8%) with NAFLD with advanced fibrosis. Patients with CAD 3 had higher median CAP-values (273±61 vs. 260±66 dB/m; p = 0.038) and higher degrees of steatosis as compared to patients without CAD 3. While NAFLD was significantly more often diagnosed in patients with CAD 3 (75.0% vs. 63.1%, p = 0.0068), no significant difference was found for NAFLD with advanced fibrosis (10.7% vs. 12.5%, p = 0.60).

**Conclusions:**

Clinically relevant CAD is frequently associated with the presence of NAFLD, but not NAFLD with advanced fibrosis.

## Introduction

Non-alcoholic fatty liver disease (NAFLD), considered to be the hepatic manifestation of metabolic syndrome (MS), is the most common liver disorder in industrialized countries and it is associated with a significantly increased risk for coronary artery disease (CAD) [1, 2]. A high prevalence of hepatic steatosis has been detected in patients who underwent invasive coronary angiography with significantly increased prevalence of relevant CAD in patients with steatosis compared to those who did not display signs of hepatic lipid accumulation on conventional ultrasound [3]. NAFLD is defined as lipid accumulation in ≥5% of hepatocytes in liver histology [4]. In conventional B-mode ultrasound (US), steatosis can only be detected if >30% of hepatocytes are affected by lipid accumulation with a sensitivity and specificity of >80% [5, 6]. A newer ultrasound based method named controlled attenuation parameter (CAP), which is integrated in the FibroScan® machine, allows quantification of steatosis if ≥10% of hepatocytes are affected and studies have reported diagnostic accuracy of 80–90% [7].

NAFLD is a risk factor for the development of non-alcoholic steatohepatitis (NASH). NASH is characterized by fibrotic remodeling of the steatotic liver parenchyma [4]. Patients with NASH are at risk to progress to liver cirrhosis with all its associated complications including hepatocellular carcinoma (HCC) and a significantly increased liver-associated mortality compared to NAFLD [2].

Furthermore, patients with NAFLD have a significantly higher risk for higher-grade CAD than patients without liver steatosis, even after adjustment for various demographic and metabolic parameters [8].

Several studies have shown that ultrasound-based transient elastography (TE, FibroScan®, Echosens, Paris, France) detects significant hepatic fibrosis with a diagnostic accuracy >80%, hence allowing for the differentiation between NAFLD with advanced fibrosis and NAFLD if applied together with CAP [2, 9].

In our prospective study, patients presenting for elective invasive coronary angiography received a FibroScan examination (TE + CAP) as well as blood analysis for biomarkers of hepatic fibrosis and elevated cardiovascular risk, respectively. The aim of this study was to investigate the association of coronary artery disease and NAFLD/ NAFLD with advanced fibrosis defined by non-invasive assessment of liver steatosis and stiffness using the FibroScan.

## Patients and methods

### Study cohort

Informed consent was obtained from all participants and the study protocol was approved by the ethical committee of the University of Frankfurt. The study was registered with Clinical-trials.com with the registration number NCT01638832.

We conducted a single-blinded prospective mono-center study in which 576 consecutive patients who presented to the cardiology department of the University Hospital Frankfurt to undergo elective coronary angiography for various indications between January 2012 and October 2014 were enrolled. Patients were excluded if they had ascites, were pregnant or breastfeeding, age < 18 years, or absence of full legal capacity. In addition to the exclusion criteria registered at Clinical-trials.com, patients with an acute myocardial infarction were excluded due to logistical reasons- transient elastography was not available on ICU ward for these patients. Patients with cardiac pacemaker or implantable cardioverter defibrillator (ICD) needed to undergo a pacemaker check immediately before and after TE as a safety precaution. Patients with clinically apparent cirrhosis (elevated bilirubin and reduced platelet count) were not excluded from the present study, if they did not have ascites.

### Coronary angiography

Standard selective coronary angiography was performed by an experienced cardiologist without knowledge about the laboratory data or TE/CAP results of the patients. The most common indications were suspected first-time manifestation of CAD and suspected progression of known CAD, respectively.

The number of diseased vessels was identified according to the number of major coronary arteries having ≥75% stenosis. Coronary Artery Disease was graded as follows: CAD 0 = no coronary artery disease, CAD 1 = mild CAD with wall irregularities exclusively, CAD 2 = moderate CAD with stenosis less than 5% reduction of the luminal diameter, CAD 3 = relevant high-grade CAD promoting stenosis with at least 75% reduction of the luminal diameter with need for coronary revascularization [10]. The decision on percutaneous coronary intervention, medical or surgical therapy was made according to clinical presentation and angiographic findings.

### Transient elastography and controlled attenuation parameter

Ultrasound-based assessment for steatosis and fibrosis was performed using a FibroScan® 502 touch device (Echosens, Paris, France). FibroScan® is a device developed specifically to quantify liver stiffness using the transient elastography technique. A vibration generator which is integrated into the ultrasound probe sends a vibration impulse into the liver tissue. The propagation velocity of the impulse which is positively correlated with the stiffness of the liver tissue is detected by ultrasound signals. This allows for the quantification of liver stiffness which is in turn associated with the degree of hepatic fibrosis if certain potential confounders are excluded. Liver stiffness is measured in kPa with a maximum value of 75 kPa [11–14].

Patients were measured after overnight fasting. Examiners were trained and certified by Echosens before participating in the study. The examination was performed on the right lobe of the liver through the intercostal space with the FibroScan® M-Probe (3.5 MHz; shear wave frequency 50 Hz; depth of measurement 25–65 mm) using the FibroScan® 502 touch device (Echosens, Paris, France). Patients were lying in a supine position. After the area of measurement was located, the examiner pressed the button of the probe to start the acquisition. At least ten valid TE measurements were required with an interquartile range (IQR) of ≤30% in patients with TE values >7.1 kPa [15]. In the present study a TE cutoff value of 7.9 kPa was defined to detect liver fibrosis stage ≥F3 [16].

Controlled attenuation parameter (CAP) is a rather novel method to quantify the attenuation of ultrasound waves in the course of tissue penetration. In conventional B-mode sonography, this gradual attenuation of ultrasound, which occurs in an accentuated manner in steatotic liver tissue, can only be assessed subjectively by the observer. CAP allows for the quantification of this phenomenon on a dB/m scale by measuring radio frequency signal attenuation in defined depths of liver tissue using a specific algorithm during regular TE with a 3.5-MHz probe. The newer generation of FibroScan devices is equipped with this technique in addition to regular TE so that CAP can be performed without extra time or effort [17]. During study performance CAP was not available for the XL-probe [18].

In the present study, hepatic steatosis using CAP was defined using cutoff values as derived from Karlas et al of 234 dB/m, 269 dB/m and 301 dB/m for S1, S2 and S3 steatosis, respectively [19].

NAFLD was determined by CAP ≥234 dB/m. NAFLD with advanced fibrosis was determined by TE-values ≥7.9kPa in the presence of NAFLD and absence of congestive or right-sided heart failure.

#### Laboratory testing and metabolic syndrome

Venous blood samples were taken for basic blood count, coagulation parameters including fibrinogen, fasting glucose, triglycerides, cholesterol, NT-proBNP, troponin T, total bilirubin, ALT, AST, GGT, HbA1c, alpha2-macroglobulin, haptoglobin, and apolipoprotein A1. The serum marker FibroMAX (BioPredictive S.A.S., Paris, France) was performed as far as laboratory values for calculation were available. FibroMAX includes the serum fibrosis marker FibroTest, the serum inflammatory marker ActiTest, the steatosis marker SteatoTest, the Nash marker NashTest. FibroMax combines the ten standard biomarkers: gamma-GT, total bilirubin, alpha-2-macroglobulin, apolipoprotein A1, haptoglobin, alanine aminotransferase (ALT), AST transaminase, triglycerides, cholesterol, fasting glucose. These markers are weighted according to the patient's age, sex, weight, and height.

Patients were classified as having metabolic syndrome (MS) if the AHA/NHLBI criteria were met: central obesity plus any two of the following four factors: raised triglycerides (≥1.7 mmol/L) or specific treatment, reduced HDL cholesterol (< 1.03 mmol/L in males, < 1.29 mmol/L in females) or specific treatment, raised blood pressure (systolic BP ≥ 130 or diastolic BP ≥ 85 mmHg) or specific treatment, raised fasting plasma glucose (≥ 5.6 mmol/L) or previously diagnosed type 2 diabetes.

#### Sample size calculation

Primary study aim was the assessment of NAFLD prevalence with and without advanced fibrosis in a patient population with indication for coronary angiography using TE and CAP. According to the results of the study by Wong et al. [3], we assumed a NAFLD prevalence of 60% in the study population with a 30% risk of progression to advanced NAFLD fibrosis [2]. A confidence interval length of approx. 10% or less for the prevalence of NAFLD with advanced fibrosis within the NAFLD subpopulation can be achieved if at least 343 patients with NAFLD are available for analysis given a NAFLD with advanced fibrosisprevalence of 30% within the NAFLD subpopulation. Assuming a NAFLD prevalence of 60%, at least 572 patients in total should be included. These sample size should also allow a confidence interval length of 10% or less for the prevalence of NAFLD with and without advanced fibrosis within the group of all evaluable patients. The goal was achieved with the enrollment of 576 patients into the study. Even after excluding patients with TE measurement failure confidence interval of the prevalence is even smaller than 10%.

### Statistical analysis

Statistical analysis of the gathered data was performed using BiAS. for Windows (version 11.06, epsilon-Verlag; Hochheim, Darmstadt, Germany). Significance level was set at α = 0.05.

Analysis for independence of dichotomous nominal variables was performed using χ^2^ test without Yates’ correction and Fisher’s exact square test, respectively. Furthermore, 95% confidence intervals for prevalences are given. Ratio scale variables were compared with Wilcoxon-Mann-Whitney U test (2 groups) and described by median values or mean ± standard deviation. A possible positive trend between the CAD values concerning the CAP values was analysed by the Jonckheere-Terpstra-Test. Multiple logistic regression analysis was performed with CAD 3 resp. NAFLD as target size. Previously univariate tested variables like BMI, hip-waist-ratio, type 2 diabetes, sex distribution, FibroTest, SteatoTest, triglycerides>150mg/dl and fatty liver index which were significant are included in the regression models. All analyses are performed using BiAS (version 11.06, epsilon-Verlag; Hochheim, Darmstadt, Germany).

## Results

**Fig 1:**
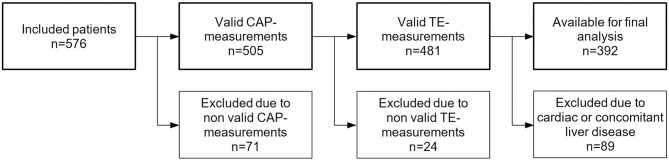
Flow chart of study inclusion.

576 consecutive patients undergoing elective coronary angiography were enrolled in the present study. 505 patients (395 males, 110 females, mean age 65.7 years) were available for analysis of NAFLD, having at least 10 successful CAP measurements. In 481 patients quality criteria for TE were met. However, in additional 88 patients, TE was faulted by congestive or right-sided heart failure (detected by echocardiography), and in one case by suspected concomitant liver disease in a patient with scleroderma. Thus, 392 patients could be evaluated for the presence of NAFLD with advanced fibrosis using TE and CAP (Fig 1). 70.5% of patients (356/505, 66.3%-74.4%) had relevant CAD (CAD grade 3, [CAD 3]), defined as stenosis with ≥75% reduction of the luminal diameter in at least one coronary vessel. Patients with CAD 3 had a higher median BMI (27.3 vs. 26.2, p = 0.05), and a longer median smoking history (16 vs. 5 pack years, p = 0.026). CAD 3 was detected significantly more often in males with indication for elective coronary angiography (302 out of 395 patients, 76.5%) compared to females (54 out of 110 patients, 49.1%) (p = <0.000001) and in type 2 diabetics (81.3% vs. 67.1%, p = 0.0023). Details on anthropometric and laboratory data are shown in Table 1 and S1 Table.

**Table 1:** Patient characteristics.

Characteristics	Included Patients		No NAFLD		NAFLD		NAFLD with advanced fibrosis	
	n = 505		n = 144		n = 361		n = 44	
age [y]	67	(18)	68	(20)	66	(18)	70	(19)
	505	[21–88]	144	[21–88]	361	[29–88]	44	[43–88]
male sex	78.2%		83.3%		76.2%		81.8%	
	395/505		120/144		275/361		36/44	
height [cm]	172	(11)	171.5	(8)	172	(12)	174	(9)
	503	[142–202]	144	[149–202]	359	[142–197]	43	[152–186]
weight [kg]	80	(20)	71	(16)	84	(21)	85	(22)
	500	[42–145]	143	[43–110]	357	[42–145]	43	[57–145]
body mass index [kg·m^-2^]	27.0	(6.4)	24.1	(4.4)	28.0	(6.0)	29.4	(8.2)
	501	[16.3–50.8]	143	[16.3–34.4]	358	[18.7–50.8]	43	[20.5–50.8]
waist circumference [cm]	101	(16)	92	(12.5)	104	(14)	108	(16.25)
	493	[63–157]	142	[69–124]	351	[63–157]	42	[78–140]
hip circumference [cm]	98	(11)	94	(10)	100	(11)	102	(11.5)
	493	[61–136]	142	[78–109]	351	[61–136]	42	[84–136]
hip-waist-ratio	0.978	(0.090)	1.012	(0.102)	0.961	(0.080)	0.945	(0.072)
	493	[0.76–1.43]	142	[0.77–1.28]	351	[0.76–1.43]	42	[0.82–1.17]
smoking history [packyears]	15	(37)	16	(41.5)	15	(35)	20	(35)
	486	[0–200]	142	[0–150]	344	[0–200]	42	[0–93]
ethanol consumption [g·d^-1^]	6	± 9	5	± 8	6	± 9	7	± 8
	485	[0–30]	142	[0–30]	343	[0–30]	41	[0–27]
heart rate [min^-1^]	70	(19)	72	(23)	70	(17)	70	(17)
	505	[37–140]	144	[37–136]	361	[40–140]	44	[51–98]
systolic blood pressure [mmHg]	129	(30)	125	(30)	130	(30)	130	(34)
	505	[80–210]	144	[60–168]	361	[85–210]	44	[85–190]
diastolic blood pressure [mmHg]	75	(15)	71	(18.5)	77	(10)	78	(14)
	505	[40–110]	144	[46–100]	361	[40–110]	44	[40–100]
LVEF [%]	55	(20)	57	(20)	55	(17)	58	(16)
	445	[10–75]	127	[10–70]	318	[13–75]	38	[13–65]
fasting glucose [mg·dl^-1^]	100	(35)	96	(26)	102	(38)	109	(38)
	493	[29–352]	141	[46–265]	352	[29–352]	44	[65–336]
triglycerides [mg·dl^-1^]	117	(87)	97	(61)	122	(97)	157	(121.75)
	460	[2–676]	131	[34–446]	329	[2–676]	42	[51–546]
total cholesterol [mg·dl^-1^]	168	(70)	156	(58)	172	(70)	166	(86)
	460	[8–392]	131	[66–336]	329	[8–392]	42	[87–340]
LDL cholesterol [mg·dl^-1^]	88	(51)	84	(47)	92	(56)	81	(64)
	449	[12–265]	129	[26–217]	320	[12–265]	41	[32.2–209]
HDL cholesterol [mg·dl^-1^]	45.8	(20.0)	48.85	(24.5)	45.2	(19.1)	45.1	(19.9)
	458	[1.9–152.2]	130	[1.9–112.0]	328	[13–152]	42	[26–109]
NT-proBNP [pg·dl^-1^]	441.7	(1432.9)	484.7	(2451.9)	430.3	(1191.2)	282.7	(1165.7)
	427	[5–70000]	115	[11–70000]	312	[5–28986]	38	[5–4203]
Troponin T [pg·dl^-1^]	13.0	(26.2)	17.7	(32.3)	12.0	(21.5)	15	(23.0)
	449	[3–6252]	122	[3–6236]	327	[3–6252]	39	[3–772]
total bilirubin [mg·dl^-1^]	0.5	(0.4)	0.5	(0.4)	0.5	(0.3)	0.6	(0.4)
	466	[0.1–4.1]	131	[0.1–2]	335	[0.1–4.1]	41	[0.2–1.4]
Gamma-glutamyl-transferase [U·l^-1^]	36	(41)	39	(48)	35	(37)	53	(81)
	437	[4–1068]	124	[9–344]	313	[4–1068]	35	[15–1068]
AST/GOT [U·l^-1^]	26	(13)	26	(12)	25	(13)	30	(30.5)
	484	[0.4–321]	138	[10–321]	346	[0.4–188]	44	[15–162]
ALT/GPT [U·l^-1^]	23	(18)	20	(17)	24	(17)	27.5	(32)
	486	[2–335]	138	[2–138]	348	[4–335]	44	[5–108]
HbA1c [%Hb]	5.8	(0.9)	5.6	(0.7)	5.8	(0.8)	6.0	(1.0)
	427	[4.6–23.0]	115	[4.8–13.1]	312	[4.6–23.0]	39	[4.88–10.2]
hemoglobin [g·dl^-1^]	13.4	(2.6)	13.1	(2.5)	13.5	(2.5)	13.5	(2.775)
	500	[7.8–19.7]	144	[8.5–16.4]	356	[7.8–19.7]	44	[9.1–15.9]
thrombocytes [nl^-1^]	217	(81)	226.5	(93)	215	(80)	192.5	(73)
	500	[20–809]	144	[33–579]	356	[20–809]	44	[80–725]

Patient characteristics are given for included patients, patients without NAFLD, with NAFLD, and NAFLD with advanced fibrosis, respectively.

Values are given as Median (inter quartile range) or Mean ± standard deviation, available data sets and [range].

### Association between CAD and NAFLD/metabolic syndrome

361 out of 505 patients (71.5%, 67.3%-75.4%) were diagnosed with NAFLD, determined by a CAP value ≥234 dB/m. There was no age difference between patients with or without NAFLD. However, BMI was higher in the NAFLD group (28.5 vs. 24.4 kg/m^2^, p<0.000001) and NAFLD was detected more frequently in patients with type 2 Diabetes compared to non-diabetics (79.5 vs. 68.5%, p = 0.018).

**Fig 2:**
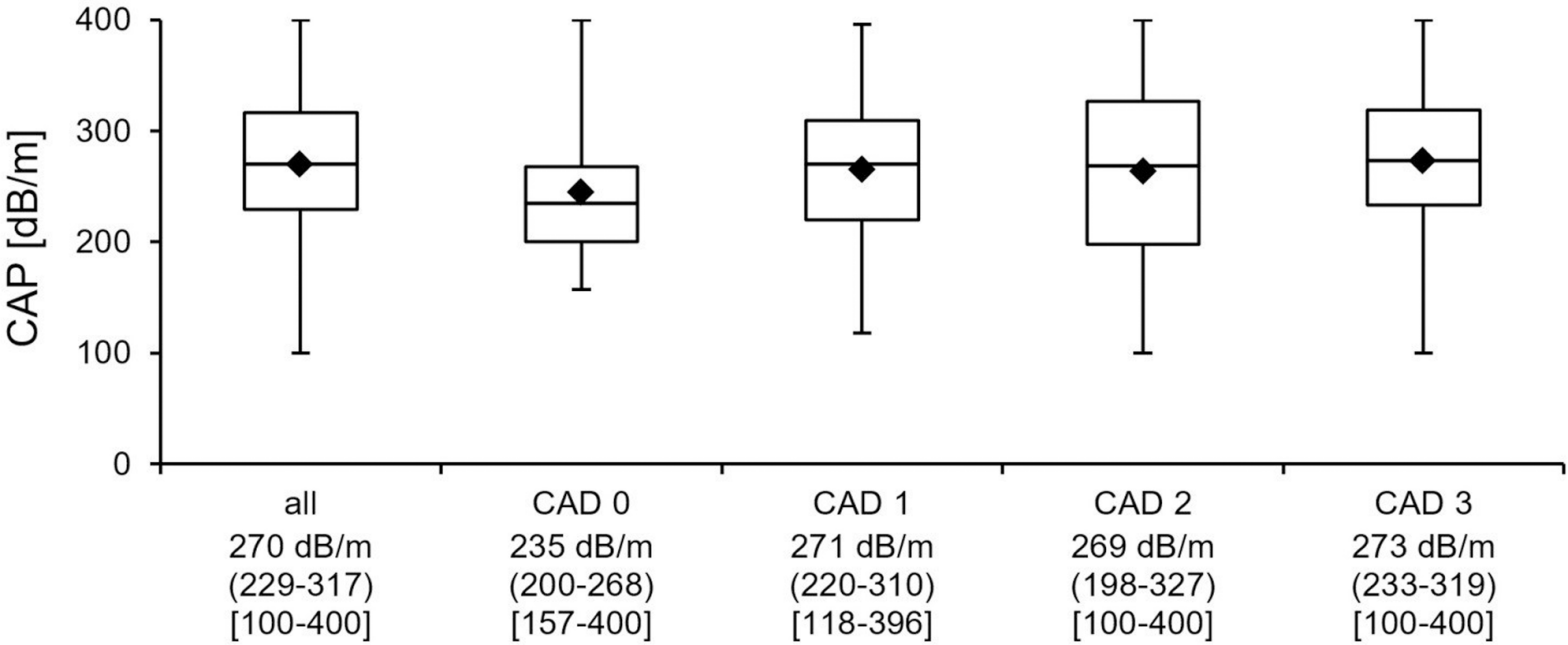
Box plot showing the association between steatosis and CAD severity. The x-axis charts shows from top row to bottom row: group name, total number or patients, median CAP-value, CAP-value in IQR (1^st^ quartile - 3^rd^ quartile), CAP-value in [range]. Abbreviations: CAP = controlled attenuation parameter, CAD 0 = no coronary artery disease, CAD 1 = plaques, CAD 2 = stenosing coronary artery disease, CAD 3 = relevant coronary artery disease.

Patients with CAD showed significantly higher CAP values compared to patients without CAD (235 for CAD 0 vs. 271 for CAD 1 vs. 269 for CAD 2 vs. 273 dB/m for CAD 3; p = 0.010) (Fig 2). In the group of patients with CAD, CAP did not differ significantly between different stages of CAD (p > 0.4). This is also true for the prevalence of NAFLD (p > 0.20). Prevalence of NAFLD was 50% in CAD 0, 66.2% in CAD 1, 65.2% in CAD 2 and 75.0% in CAD 3 (Kruskal-Wallis-Test for CAD 0–3 p = 0.019. Kruskal-Wallis-Test for CAD 1–3 p = 0.14)

NAFLD was significantly more often diagnosed in patients with CAD 3 (75.0% vs. 63.1%, p = 0.0068) and patients with CAD 3 exhibited higher degrees of steatosis than those with minor or no CAD (CAP-values 273±61 vs. 261±66 dB/m; p = 0.038). Whereas prevalence of steatosis was just 50% in patients without CAD, 72.7% of patients with any degree of CAD displayed features of NAFLD (p = 0.013).

Univariate analyses showed that patients with CAD 3 had significant higher BMI values (p = 0.026), a higher fatty liver index (p = 0.0035), and a lower hip-waist-ratio (p = 0.0019) than patients without CAD 3. Further, sex distribution (p<0.0001), nicotine consumption (p = 0.011), triglyceridas >150 mg/dl (p = 0.0024), hypertension (p = 0.015) and type 2 diabetes (p = 0.044) are significant variables. In contrast, age and metabolic syndrome do not have a significant influence on CAD 3. However in multiple logistic regression analysis only NAFLD, sex distribution, hypertension and hip-waist-ratio remained independently associated with CAD 3, see Table 2.

**Table 2:** Multiple logistic regression analysis (stepwise backward variable selection with p values <0.1) with CAD 3 as dependent variable. OR: odds ratio; 95%-CI: 95% confidence interval.

	OR	95% CI of OR	p value
Constant	56.45	2.66–1199.25	0.01
NAFLD	1.7	1.04–2.78	0.035
sex distribution	0.36	0.21–0.61	0.0001
hip-waist-ratio	0.05	0.003–0.98	0.048
arterial hypertension	2.31	1.05–5.07	0.037

A further analysis showed that NAFLD is significantly associated with BMI (p<0.0001), type 2 diabetes (p<0.0001), metabolic syndrome (p<0.0001), hip-waist-ratio (p<0.0001), triglycerides <150 g/dl (p<0.0001), fatty liver index (p<0.0001) and SteatoTest-Score (p<0.0001), but not with FibroTest-Score, sex distribution and nicotine consumption. In a multiple logistic regression analysis only BMI and metabolic syndrome remained significant, see Table 3.

**Table 3:** Multiple logistic regression analysis (stepwise backward variable selection with p values <0.1) with NAFLD as dependent variable. OR: odds ratio; 95%-CI: 95% confidence interval.

	OR	95% CI of OR	p value
Constant	0.0035	0.0004–0.03	<0.0001
BMI	1.27	1.17–1.39	<0.0001
metabolic syndrome	2.21	1.17–4.18	0.014

**Fig 3:**
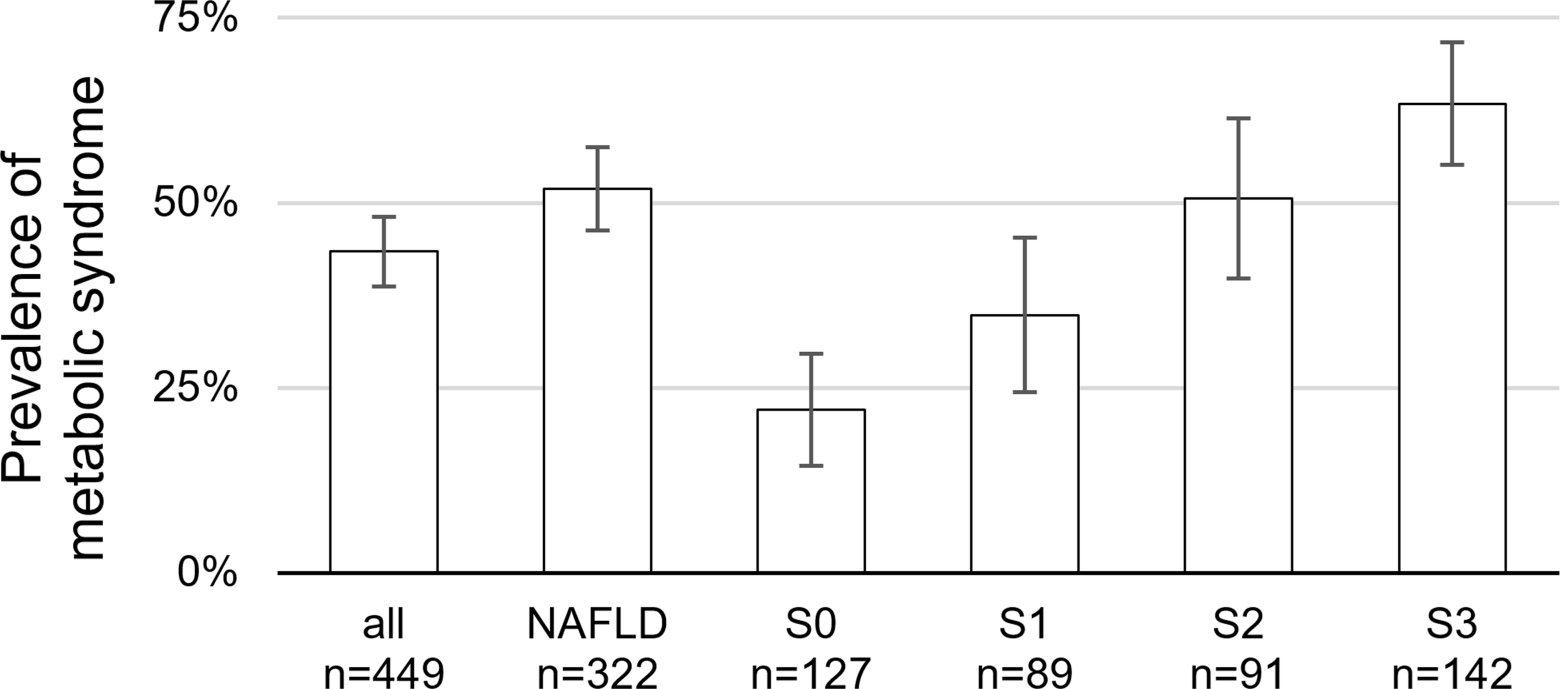
Prevalence of metabolic syndrome in association with hepatic steatosis. Abbreviations: S0 = no steatosis, S1 = steatosis of 5–33% of hepatocytes, S2 = steatosis of 33–66% of hepatocytes, S3 = steatosis of >66% of hepatocytes.

Laboratory values to define the presence or absence of metabolic syndrome was available in 449/505 patients. There was a distinct association between MS and the severity of NAFLD (22.1% of non-NAFLD-patients and 51.9% of NAFLD patients had MS, p<0.01). Within the NAFLD population, higher degree of steatosis was associated with higher prevalence of MS (Fig 3). There was a highly significant correlation between the presence of MS and CAD. Whereas just 30.4% of patients without CAD had MS, the prevalence of MS was 44.1% in patients with any grade of CAD (p<0.01) and furthermore 46.9% in the CAD 3 population requiring coronary intervention, respectively.

FibroMAX-analysis of markers of fibrosis (BioPredictive S.A.S., Paris, France) was performed in 320 patients irrespective of validity of transient elastography. No significant differences between distinct stages of CAD throughout FibroTest, ActiTest, SteatoTest, NashTest or AshTest could be found.

### Association between CAD and NAFLD with advanced fibrosis

Liver fibrosis ≥ F3 was found in 54 out of 392 assessable patients (13.8%, 10.5%-17.6%). Ten out of the 54 patients did not display signs of NAFLD, i.e. CAP <234 dB/m. The remaining 44 out of 392 patients (11.2%, 8.3%-14.8%) were classified as having NAFLD with advanced fibrosis(i.e. TE-values ≥7.9kPa in the presence of NAFLD) (Fig 3). This corresponds to 44 patients with advanced fibrosis within the group of 288 assessable NAFLD patients (15.3%, 11.1%-19.4%). Patients with NAFLD and advanced fibrosis had a higher BMI (30.2 vs. 27.1kg/sm, p = 0.00053) than individuals without advanced fibrosis.

**Fig 4:**
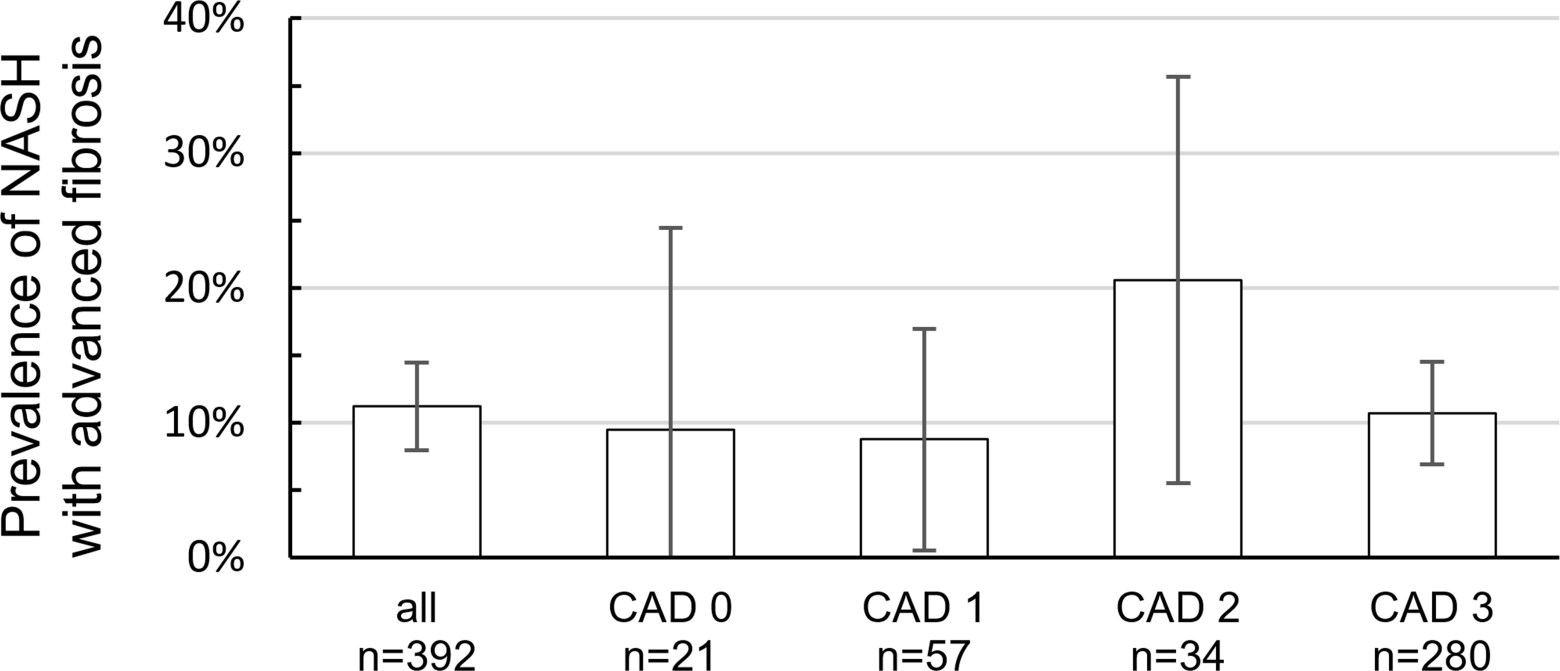
Prevalence of NAFLD with advanced fibrosis in association with CAD. No significant difference was shown between the groups with CAD 0 vs. CAD 1 (p = 1.0), CAD 0 vs. CAD 2 (p = 0.38), and CAD 0 vs. CAD 3 (p = 1.0). Abbreviations: CAD 0 = no coronary artery disease, CAD 1 = plaques, CAD 2 = stenosing coronary artery disease, CAD 3 = relevant coronary artery disease.

There was no significant difference concerning the presence of NAFLD with advanced fibrosis detected by TE between patients with and without CAD (8.8% vs. 9.5%, p = 1.0) or between patients with CAD 3 compared to those with CAD 0–2 (10.7% vs. 12.5%, p = 0.60) (Fig 4). Details on fibrosis distribution according to CAD stage and steatosis are shown in Table 4.

**Table 4:** Fibrosis distribution measured with TE according to steatosis and CAD stage.

	Included patients	Valid TE	No NAFLD	NAFLD	CAD 0	CAD 1	CAD 2	CAD 3
	n = 505	n = 392	n = 104	n = 288	n = 21	n = 57	n = 34	n = 280
Transient Elastography (TE)	5.8 (3.7)	5.3 (4.3)	5.1 (2.3)	5.4 (2.4)	0.3 (2.0)	5.7 (2.5)	5.4 (3.3)	5.3 (2.4)
	[0.3–75.0]	[0.3–35.3]	[1.5–16.8]	[0.3–35.3]	[2.8–12.0]	[0.3–35.3]	[2.3–19.6]	[1.5–26.3]
F2-Fibrosis [%]	-	108 (27.6)	-	-	4 (19.0)	20 (35.1)	6 (17.6)	78 (27.9)
F3-Fibrosis [%]	-	54 (13.8)	-	-	3 (14.3)	8 (14.0)	7 (20.6)	36 (12.9)

NAFLD with advanced fibrosis in relation to NAFLD was more common in patients without rCAD, however this difference was not significant.

Further, patients with NAFLD with advanced fibrosis showed significantly higher values concerning fatty liver index (p<0.0001), FibroTest-Score (p<0.0001) and SteatoTest-Score (p<0.0001). In addition, a significant correlation between NAFLD with advanced fibrosis and values of triglycerides > 150 mg/dl was found. Significantly more patients with NAFLD with advanced fibrosis had values of triglycerides > 150 mg/dl than NAFLD patients without advanced fibrosis (p = 0.015) and showed more often type 2 diabetes (p = 0.0096). A multiple logistic regression analysis with NAFLD with advanced fibrosis as dependent variable was not feasible here because of the small sample size.

## Discussion

Coronary artery disease and nonalcoholic fatty liver disease share common risk factors, most of all metabolic syndrome. NAFLD is particularly common in diabetic patients with a prevalence of 34–74% compared to 17–33% in the general population [1] and it is associated with a 1.44- to 2.05-fold increased risk of coronary artery disease compared to the general population [2]. While NAFLD itself is not associated with increased mortality, it represents an important risk factor for the development of non-alcoholic steatohepatitis (NASH). 10–30% of patients with NAFLD develop NASH, and again about one third of NASH patients progress to liver cirrhosis with all of its well-known detrimental effects including hepatocellular carcinoma (HCC). As a consequence, NASH is associated with a significantly increased liver-associated mortality compared to NAFLD [2]. It has remained unclear whether these pathophysiologic associations warrant a routine screening for NAFLD in all CAD patients.

Our single-blinded prospective mono-center study including 576 consecutive patients undergoing elective coronary angiography for various indications aimed to evaluate the association between CAD and NAFLD/ NAFLD with advanced fibrosis using transient elastography for the diagnosis of liver fibrosis and CAP for the diagnosis of liver steatosis. The ability of ultrasound-based transient elastography (TE) to detect hepatic fibrosis and differentiate between steatosis without advanced fibrosis (NAFLD) and NAFLD with advanced fibrosis if applied together with CAP has been shown in previous studies [2, 9]. Since TE and CAP are included in the same device (FibroScan®), they can be executed within a 5 minute, non-invasive and cost-effective examination, sparing the patient a painful and potentially dangerous liver biopsy. In the present study a TE cutoff value of 7.9 kPa was defined to detect liver fibrosis. This cutoff value has previously been shown to detect ≥F3 fibrosis with a sensitivity, specificity, and positive and negative predictive values of 91%, 75%, 52%, and 97%, respectively [16]. Hepatic steatosis was detected using CAP cutoff values of 234 dB/m, 269 dB/m and 301 dB/m for S1, S2 and S3 steatosis, respectively, as derived from Karlas et al. [19]. Applying the CAP method enables the detection of hepatic steatosis in patients with as little as 10% of hepatocytes affected with 80–90% diagnostic accuracy. This explains the high yield of 71.5% NAFLD prevalence across our entire cohort as compared to a similar population assessed exclusively with conventional ultrasound which found a NAFLD prevalence of 58.2% (356/612) [20].

In the present study, we detected a high incidence of NAFLD (72.7%) in patients with any grade of CAD (compared to 50% in patients without any CAD) with even higher rates in individuals with severe CAD. Patients with CAD 3 exhibited higher degrees of steatosis than those with minor or no CAD (p = 0.038). This is in accordance with a previous study by Wong et al. [3], who have used ultrasound for the detection of liver steatosis. Wong et al. [3] have assessed 612 patients who underwent invasive coronary angiography for sonographically evident hepatic steatosis. 58% of patients have had sonographical signs of steatosis. CAD has been found in 85% of patients with steatosis detected by ultrasound as compared to 64% of patients without steatosis (p<0.001). Liver steatosis has remained an independent risk factor for CAD even after adjusting for demographic and metabolic parameters (odds ratio (OR) 2.31) [3]. However, somewhat contrary to our expectations, there has been no significant difference in the prevalence of NAFLD with advanced fibrosis between patients with and without CAD (8.8% vs. 9.5%, p = 1.0) or between patients with CAD 3 compared to those with CAD 0–2 (10.7% vs. 12.5%, p = 0.60). One possible reason could be, that cardiovascular complications occur much earlier in NAFLD patients and therefore outweigh potential hepatic complications such as hepatocellular carcinoma and cirrhosis [20]. However, long-term follow-up studies are needed for further clarification.

A limitation of the present study is the high failure rate of transient elastography (16.5%). At the time of study performance only the FibroScan® M-probe included the CAP-technique. However, since the assessment of liver steatosis was essential for the diagnosis of NAFLD, this probe was chosen. The failure rate was in accordance with previous studies using the M-probe [21, 22]. In the mean time, CAP is available for the XL-probe which is associated with reduced failure rate [8, 18].

To avoid known potential confounders such as food intake or upright position, the measurements in the present study were performed during fasting in a standardized supine position [23]. Congestive or isolated right-sided heart failure are well-known to affect liver stiffness and produce increased TE values.

TE values increase after meal intake [24], TE was therefore performed fasting in the present study. Factor increasing tension within the Glisson’s capsule also increase TE values, leading to an overestimation of liver fibrosis. Such factors include necroinflammatory histological activity; alanine aminotransferase flare in acute or chronic hepatitis; elevated central venous pressure; cardiac failure and intrahepatic or extrahepatic cholestasis; and excessive alcohol intake [25–30]. Necroinflammatory histology was not available in the present study. After stratification for ALT-concetration no significant difference in liver stiffness could be found throughout different severities of CAD in the subgroup with normal ALT levels.

Recent data have even shown that liver elastography is a valid tool to assess for right atrial pressure or central venous pressure in congestive heart failure or congenital heart disease and might even be superior to echocardiography to evaluate right-sided congestion [31–33]. We therefore had to additionally exclude 88 patients from our final analysis who formally met the TE quality criteria but displayed echocardiographical signs of heart failure from our final analysis.

A recent study by Wong et al. reports a lower validity of CAP for the diagnosis of NAFLD if the IQR of CAP is ≥40dB/m [34]. In the present study this was the case in 27% of patients. Stratification for CAP measurements with less than 40 dB/m IQR still showed a significant increase in prevalence of metabolic syndrome and relevant coronary artery disease with higher grades of steatosis.

Another limitation is, that laboratory values were missing in 10% of patients. Albumine was a missing value in the present study, therefore NAFLD fibrosis score–as recommended in recent guidelines [13] could not be calculated.

Patients with reported acute or chronic liver disease were excluded from the present study. Nevertheless, patients with undiagnosed liver disease could have been missed. 10 patients had advanced fibrosis without steatosis, concomittant liver diesease in these patients is suggestive. Nevertheless these patients dropped out from patients with „NAFLD and advanced fibrosis”and therefor did not influence the prinary study analysis. None of the patients took medications that were known to modulate the degree of hepatic steatosis (e.g. vitamin E).

In summary, the present study reveals that clinically relevant CAD is frequently associated with the presence of NAFLD. However, the risk for NAFLD with advanced fibrosis in these patients is low.

## Supporting information

S1 Table Detailed patient characteristics. Patient characteristics are given for included patients, patients without and with NAFLD, NAFLD with advanced fibrosis, without and with relevant CAD. Values are given as Median (inter quartile range) or Mean ± standard deviation, available data sets and [range].
(DOCX)
