# Supplementary material for: Severity of coronary artery disease is associated with non-alcoholic fatty liver dis-ease: A single-blinded prospective mono-center study
Source: PLoS One. 2017 Oct 26;12(10):e0186720. doi: 10.1371/journal.pone.0186720 (PMC5658076; doi:10.1371/journal.pone.0186720)
Supplement: S1 Table — Patient characteristics are given for included patients, patients without and with NAFLD, NAFLD with advanced fibrosis, without and with relevant CAD. Values are given as Median (inter quartile range) or Mean ± standard deviation, available data sets and [range]. (DOCX) [file pone.0186720.s001.docx]

**Supplementary Table 1: Detailed patient characteristics**

| Characteristics | Included Patients | | No NAFLD | | NAFLD | | NAFLD with advanced fibrosis | | No relevant CAD | | Relevant CAD | |
| --- | --- | --- | --- | --- | --- | --- | --- | --- | --- | --- | --- | --- |
|  | n=505 | | n=144 | | n=361 | | n=44 | | n=149 | | n=356 | |
| age [y] | 67 | (18) | 68 | (20) | 66 | (18) | 70 | (19) | 64 | (21) | 67 | (17) |
|  | 505 | [21-88] | 144 | [21-88] | 361 | [29-88] | 44 | [43-88] | 149 | [21-88] | 356 | [34-88] |
| male sex | 78.2 % | | 83.3 % | | 76.2 % | | 81.8 % | | 62.4 % | | 84.8 % | |
|  | 395/505 | | 120/144 | | 275/361 | | 36/44 | | 93/149 | | 302/356 | |
| height [cm] | 172 | (11) | 171.5 | (8) | 172 | (12) | 174 | (9) | 172 | (13) | 172 | (10) |
|  | 503 | [142-202] | 144 | [149-202] | 359 | [142-197] | 43 | [152-186] | 148 | [142-202] | 355 | [144-197] |
| weight [kg] | 80 | (20) | 71 | (16) | 84 | (21) | 85 | (22) | 77 | (25) | 80 | (19) |
|  | 500 | [42-145] | 143 | [43-110] | 357 | [42-145] | 43 | [57-145] | 147 | [50-120] | 353 | [42-145] |
| body mass index [kg·m^-2^] | 27.0 | (6.4) | 24.1 | (4.4) | 28.0 | (6.0) | 29.4 | (8.2) | 26.2 | (7.4) | 27.3 | (6.1) |
|  | 501 | [16.3-50.8] | 143 | [16.3-34.4] | 358 | [18.7-50.8] | 43 | [20.5-50.8] | 147 | [16.3-41.7] | 354 | [18.4-50.8] |
| waist circumference [cm] | 101 | (16) | 92 | (12.5) | 104 | (14) | 108 | (16.25) | 98 | (21.5) | 101 | (14) |
|  | 493 | [63-157] | 142 | [69-124] | 351 | [63-157] | 42 | [78-140] | 144 | [63-130] | 349 | [73-157] |
| hip circumference [cm] | 98 | (11) | 94 | (10) | 100 | (11) | 102 | (11.5) | 98 | (12) | 98 | (10.5) |
|  | 493 | [61-136] | 142 | [78-109] | 351 | [61-136] | 42 | [84-136] | 144 | [78-136] | 349 | [61-130] |
| hip-waist-ratio | 0.978 | (0.090) | 1.012 | (0.102) | 0.961 | (0.080) | 0.945 | (0.072) | 1.000 | (0.124) | 0.971 | (0.079) |
|  | 493 | [0.763-1.429] | 142 | [0.768-1.275] | 351 | [0.763-1.429] | 42 | [0.823-1.172] | 144 | [0.823-1.429] | 349 | [0.763-1.288] |
| smoking history [packyears] | 15 | (37) | 16 | (41.5) | 15 | (35) | 20 | (35) | 5 | (30) | 16 | (38) |
|  | 486 | [0-200] | 142 | [0-150] | 344 | [0-200] | 42 | [0-93] | 143 | [0-192] | 343 | [0-200] |
| ethanol consumption [g·d^-1^] | 6 | ± 9 | 5 | ± 8 | 6 | ± 9 | 7 | ± 8 | 5 | ± 9 | 6 | ± 8 |
|  | 485 | [0-30] | 142 | [0-30] | 343 | [0-30] | 41 | [0-27] | 143 | [0-30] | 342 | [0-30] |
| heart rate [min^-1^] | 70 | (19) | 72 | (23) | 70 | (17) | 70 | (17) | 72 | (21) | 69 | (17) |
|  | 505 | [37-140] | 144 | [37-136] | 361 | [40-140] | 44 | [51-98] | 149 | [37-140] | 356 | [44-136] |
| systolic blood pressure [mmHg] | 129 | (30) | 125 | (30) | 130 | (30) | 130 | (34) | 130 | (35) | 129 | (30) |
|  | 505 | [80-210] | 144 | [60-168] | 361 | [85-210] | 44 | [85-190] | 149 | [80-185] | 356 | [60-210] |
| diastolic blood pressure [mmHg] | 75 | (15) | 71 | (18.5) | 77 | (10) | 78 | (14) | 75 | (12) | 75 | (13) |
|  | 505 | [40-110] | 144 | [46-100] | 361 | [40-110] | 44 | [40-100] | 149 | [42-110] | 356 | [40-110] |
| LVEF [%] | 55 | (20) | 57 | (20) | 55 | (17) | 58 | (16) | 60 | (15) | 55 | (15) |
|  | 445 | [10-75] | 127 | [10-70] | 318 | [13-75] | 38 | [13-65] | 129 | [13-75] | 316 | [10-70] |
| fasting glucose  [mg·dl^-1^] | 100 | (35) | 96 | (26) | 102 | (38) | 109 | (38) | 97 | (32) | 101 | (37) |
|  | 493 | [29-352] | 141 | [46-265] | 352 | [29-352] | 44 | [65-336] | 145 | [46-300] | 348 | [29-352] |
| triglycerides [mg·dl^-1^] | 117 | (87) | 97 | (61) | 122 | (97) | 157 | (121.75) | 109 | (76.5) | 118 | (90) |
|  | 460 | [2-676] | 131 | [34-446] | 329 | [2-676] | 42 | [51-546] | 134 | [20-499] | 326 | [2-676] |

| Characteristics | Included Patients | | No NAFLD | | NAFLD | | NAFLD with advanced fibrosis | | No relevant CAD | | Relevant CAD | |
| --- | --- | --- | --- | --- | --- | --- | --- | --- | --- | --- | --- | --- |
|  | n=505 | | n=144 | | n=361 | | n=44 | | n=149 | | n=356 | |
| total cholesterol [mg·dl^-1^] | 168 | (70) | 156 | (58) | 172 | (70) | 166 | (86) | 174 | (63) | 166 | (71) |
|  | 460 | [8-392] | 131 | [66-336] | 329 | [8-392] | 42 | [87-340] | 134 | [64-392] | 326 | [8-344] |
| LDL cholesterol [mg·dl^-1^] | 88 | (51) | 84 | (47) | 92 | (56) | 81 | (64) | 92 | (47) | 86 | (53) |
|  | 449 | [12-265] | 129 | [26-217] | 320 | [12-265] | 41 | [32.2-209] | 131 | [34-262] | 318 | [12-265] |
| HDL cholesterol [mg·dl^-1^] | 45.8 | (20.0) | 48.85 | (24.5) | 45.2 | (19.1) | 45.1 | (19.9) | 50.1 | (25.1) | 44.3 | (18.0) |
|  | 458 | [1.9-152.2] | 130 | [1.9-112.0] | 328 | [12.8-152.2] | 42 | [25.9-109.0] | 132 | [16.4-116.5] | 326 | [1.9-152.2] |
| NT-proBNP [pg·dl^-1^] | 441.7 | (1432.9) | 484.7 | (2451.9) | 430.3 | (1191.2) | 282.7 | (1165.7) | 456.2 | (1447.2) | 436.4 | (1389.3) |
|  | 427 | [5-70000] | 115 | [11-70000] | 312 | [5-28986] | 38 | [5-4203] | 126 | [5-70000] | 301 | [8-70000] |
| Troponin T [pg·dl^-1^] | 13.0 | (26.2) | 17.7 | (32.3) | 12.0 | (21.5) | 15 | (23.0) | 10.8 | (16.0) | 14.8 | (31.0) |
|  | 449 | [3-6252] | 122 | [3-6236] | 327 | [3-6252] | 39 | [3-772] | 127 | [3-364] | 322 | [3-6252] |
| total bilirubin  [mg·dl^-1^] | 0.5 | (0.4) | 0.5 | (0.4) | 0.5 | (0.3) | 0.6 | (0.4) | 0.5 | (0.4) | 0.5 | (0.3) |
|  | 466 | [0.1-4.1] | 131 | [0.1-2] | 335 | [0.1-4.1] | 41 | [0.2-1.4] | 135 | [0.1-2.8] | 331 | [0.1-4.1] |
| Gamma-glutamyl-transferase [U·l^-1^] | 36 | (41) | 39 | (48) | 35 | (37) | 53 | (81) | 38 | (46) | 36 | (37) |
|  | 437 | [4-1068] | 124 | [9-344] | 313 | [4-1068] | 35 | [15-1068] | 127 | [9-472] | 310 | [4-1068] |
| AST/GOT [U·l^-1^] | 26 | (13) | 26 | (12) | 25 | (13) | 30 | (30.5) | 25 | (12) | 26 | (13) |
|  | 484 | [0.4-321] | 138 | [10-321] | 346 | [0.4-188] | 44 | [15-162] | 141 | [12-188] | 343 | [0.4-321] |
| ALT/GPT [U·l^-1^] | 23 | (18) | 20 | (17) | 24 | (17) | 27.5 | (32) | 24 | (19) | 23 | (17) |
|  | 486 | [2-335] | 138 | [2-138] | 348 | [4-335] | 44 | [5-108] | 141 | [5-335] | 345 | [2-212] |
| HbA1c [%Hb] | 5.8 | (0.9) | 5.6 | (0.7) | 5.8 | (0.8) | 6.0 | (1.0) | 5.6 | (0.6) | 5.8 | (0.9) |
|  | 427 | [4.6-23.0] | 115 | [4.8-13.1] | 312 | [4.6-23.0] | 39 | [4.88-10.2] | 126 | [4.6-9.6] | 301 | [4.9-23.0] |
| hemoglobin [g·dl^-1^] | 13.4 | (2.6) | 13.1 | (2.5) | 13.5 | (2.5) | 13.5 | (2.775) | 13.2 | (2.4) | 13.4 | (2.6) |
|  | 500 | [7.8-19.7] | 144 | [8.5-16.4] | 356 | [7.8-19.7] | 44 | [9.1-15.9] | 147 | [7.8-19.7] | 353 | [8.4-18.0] |
| thrombocytes [nl^-1^] | 217 | (81) | 226.5 | (93) | 215 | (80) | 192.5 | (73) | 222 | (82) | 214 | (82) |
|  | 500 | [20-809] | 144 | [33-579] | 356 | [20-809] | 44 | [80-725] | 147 | [33-579] | 353 | [20-809] |

*Patient characteristics are given for included patients, patients without and with NAFLD, NAFLD with advanced fibrosis, without and with relevant CAD.*

*Values are given as Median (inter quartile range) or Mean ± standard deviation, available data sets and [range].*
